# The gut microbial diversity of colon cancer patients and the clinical significance

**DOI:** 10.1080/21655979.2021.1972077

**Published:** 2021-09-23

**Authors:** Tengfei He, Xiaohui Cheng, Chungen Xing

**Affiliations:** Department of Genenal Surgery, The Second Affiliated Hospital of Soochow University, Suzhou, China

**Keywords:** Colon cancer, microbial diversity, harmful bacteria, beneficial bacteria

## Abstract

The microbial diversity and communities in the excrement of healthy and patients suffered from cancer were identified by 16SrDNA sequencing performed on the Illumina Hi Seq sequencing platform. The microbial difference was also analyzed. The sequencing results showed high quality of the data, and the microbial communities were more various in the excrement of cancer patients. And the abundance of Firmicutes phylum was significantly reduced in cancer group. The phylum of Fermicutes, Bacteroidetes in cancer group are significantly down-regulated and up-regulated compared with normal group. The species of *Faecalibacterium prausnitzii, Bateroides vulgatus* and *Fusicatenibacter saccharivorans* are significantly lower in cancer group than that in normal group (*P*< 0.05). The species of *Prevetella copri, M. uniformis,* and *Escherichia coli* are significantly higher in the cancer group than that in normal group. The comparative results indicated that beneficial bacterium significantly decreased in colorectal cancer (CRC) group, and harmful bacterium significantly increased in the colon cancer group, meanwhile the acidity, sugar increased whereas the oxygen content decreased to facilitate the growth of harmful bacterium. The results would provide microbial approaches for the treatment of colon cancer by the intake of beneficial microbial communities.

## 1. Introduction

As the development of microbiome, more and more microbes were excavated from the gut of health crowd and patients suffered from cancer and other metabolic diseases such as Diabetes, gout, osteoporosis, vitamin D deficiency, hyperlipidemia [1–3]. And the great difference of microbial diversity and communities in healthy crowd and patients were demonstrated. And gut microbes played an important role in the development and progress of diseases [4,5]. The physiological function of gut microbial communities is closely associated with the human health. It was reported that the alteration of the microbial communities have a close relationship with the infection of human papillomavirus [6]. *Fusobacterium hwasooki* and *Porphyromonas gingivalis* were reported as harmful gut microbial that play a role in the occurrence and the development of colorectal cancer (CRC). Researchers at Harvard Medical School and the Jocelyn Diabetes Center have analyzed the genetic makeup of bacteria in the human gut, we also looked at the bacterial genome (genetic characteristics) in relation to arteriosclerosis cardiovascular disease, cirrhosis, inflammatory bowel disease, colorectal cancer, and Type 2 diabetes. Data from microbiome-disease Association studies at the genetic level suggest that coronary artery disease, IBD, and cirrhosis share many of the same bacterial genes. In other words, people whose Gut Microbiota contains the same collection of bacteria seem to be more likely to have one or more of these three conditions. Recent research suggests that microbes in the human gut may play a role in everything from obesity to cancer [7–9]. It was reported that anti-inflammatory factors, compounds with analgestic activity such as γ-aminobutyric (GABA), antioxidants and vitamins can be produced by gut microbes to protect human body. Meanwhile, some prebiotics can also yield antibiotics to inhibit the growth of harmful bacteria that can produce toxins causing chronic disease [7–9].

There are differences in the number, structure, abundance, and physiological state of microbes among individuals [10]. *Bacteroides* and *Firmicutes* sp. are the most common among the normal gut microbes, which accounted for 90% [11], and other fewer microbes were actinomycetes [12] and proteobacteria [13], etc. Gut microbes can live in different parts of human beings. And the metabolism of specific microbes and thereof produced metabolites can affect the balance of intestinal environment. Meanwhile, there is a close and mutually beneficial symbiosis between the intestinal microbes and the host. In turn, the host can also affect the communities and function of gut microbes [14,15]. The gut microbial communites of C57BL/6 J mice with high-fat diet were also significantly altered by calcium supplement [16]. Colorectal is an important digestive organ in human body, which has the function of digestion and nutrition intake. It also play the role of metabolism and the storage of food residues. However, the residue and some acids, phenols, and other carcinogens produced by metabolism can be the pathogen for intestinal [17–19]. Thus, the integrity of the barrier for the intestinal, as well as the immune system, etc., would be invaded and destroyed, and the risk of exposure would increase [20–22]. Colorectal cancer is a common type of loss of body mass. Chronic and recurrent elimination of mild and severe diarrhea and abdominal pain [23–25], which usually occurs in the ileum, colon, and rectum. The successful inoculation of gut microbiota to C57BL/6 mice administrated with antibiotics ahead was performed, thus resulting in the transmission of obese mice to lean mice. The results suggested the important physilogical role of gut microbes for hosts [26–28].

In this study, the microbial diversity and composition of the excrement from 73 healthy crowd and 60 patients suffered from colon cancer were analyzed by 16SrDNA sequencing on Illumina sequencing platform. The sequencing quality and composition, diversity of gut microbial were also analyzed. The microbial diversity and abundance of the fecal sample from healthy people and CRC patients were firstly analyzed, thus providing clues for the prevention and treatment of colon cancer by the inoculation of beneficial microbes and reducing the abundance of harmful bacteria.

## 2. Materials and methods

### 2.1. The patients and groups

61 patients and 72 normal crowds were divided into two groups. The excrement of the two individual groups were collected.

### 2.2. The DNA extraction

The DNAs of gut microbes from the excrement of different groups were extracted using the genome DNA extraction kit (Umagen, Guangzhou), and then stored in −80°C until using.

### 2.3. The 16SrDNA sequencing

DNA extracted from the fecal samples was used to amplify the V3-V4 region of 16A rRNA gene to determine the gut bacterial community structure. Primer set 341 F (5ʹ-ACTCCTCCGGGAGGCAGCAG-3ʹ) /806 R (5ʹ-GGACTACGCGGGTATCTAAT-3ʹ) using prime STAR HS mix (Takara, Japan) was employed to target the V3-V4 region. And the amplification condition was as following: Pre-denaturation at 95° C 3 min; 95° C denaturation 30 s 55° C annealing 30 s 72° C extension 30 s. A total of 29 cycles, 72° C extension for 5 min, 4° C storage. The amplified products were further subjected to library preparation and sequencing on the Illumina MiSeq platform as per the manufaturer’ s instructions (Illumina Technologies, USA).

### 2.4. The data analysis

The raw fastq files obtained by Illumina sequencing machine (Illumina Hiseq2500, USA) were quality-filtered using the Trimmomatic, vsearch, etc. The high quality sequence were used for community structure analysis using QIIME pipeline. Operational taxonomic unit (OTU) picking method was carried out using UCLUST closed reference method, and the representative OTUs were assigned taxonomy using UCLUST classifier with SILVA database (version 132) as reference dataset. Alpha and beta diversity analysis were performed, and further statistical analysis was carried out using R.

### 2.5. Dilution curve and relative abundance analysis of species.

Random sampling of OTU sequences and analysis of sequence numbers and OTU numbers were performed to prepare the dilution curve and to analyze the relative abundance of species.

### 2.6. Analysis of the composition of intestinal microbial colonies

Using Qiime software, and according to the results of OTU classification, the intestines of mice in each group were compared. The composition of trace microorganisms was analyzed, which were classified from phylum, family, genus and so on to understand the changes of the composition and structure of intestinal microorganisms in each group.

### 2.7. Similarity analysis between groups

Principal coordinate analysis (principal co-ordinates analysis,PCoA). It is a method to study the similarity between data by analyzing the distance and matrix of data. The visualization method of difference. All samples were obtained by UniFrac analysis. distance, matrix data, and then PCoA, to understand the intestinal microcosm of each group of mice to investigate the similarity between biological communities.

## 3. Results and discussion

### 3.1. Analysis of relative abundance of species

The relative abundance of species were analyzed based on the dilution curve and OTU data.

#### 3.1.1. Dilution and abundance curve analysis

**Figure 1.**
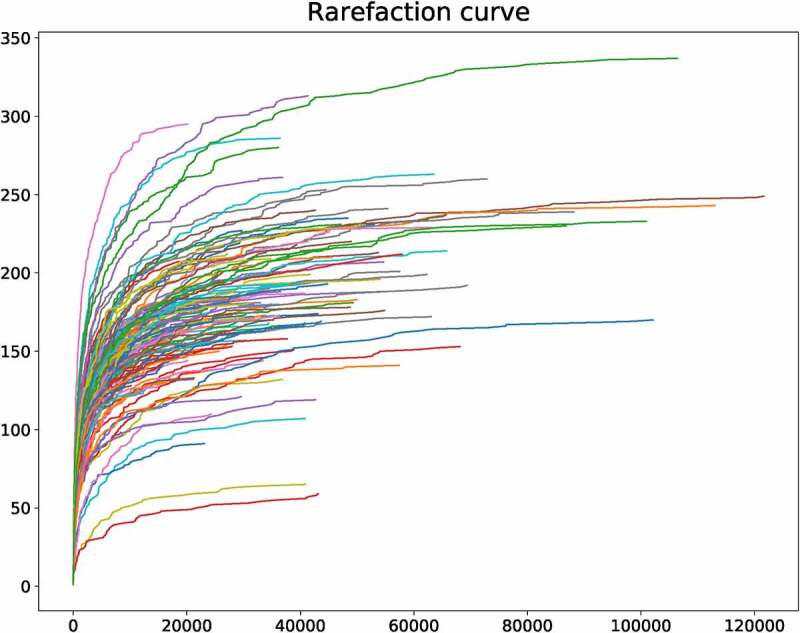
The dilution curve of 16SrRNA sequencing

The sequencing data indicated that the lengths of most reads are 450–500 bp (Fig. S1). The dilution curve can directly reflect the rationality of the collected sample. And the collected samples are enough to reflect the microbial diversity (Figure 1). The relative abundance curve was also depicted, which can reflect the abundance and uniformity of sequencing. Abscissa indicates that the relative abundance of OTU is arranged in descending order. The ordinate represents the relative abundance of the sequence number in the OTU. The species sequence number of sequence samples is mainly distributed in the range of 2000 to 8000, and the composition and distribution are evenly distributed.

#### 3.1.2. Microbial diversity analysis

**Figure 2.**
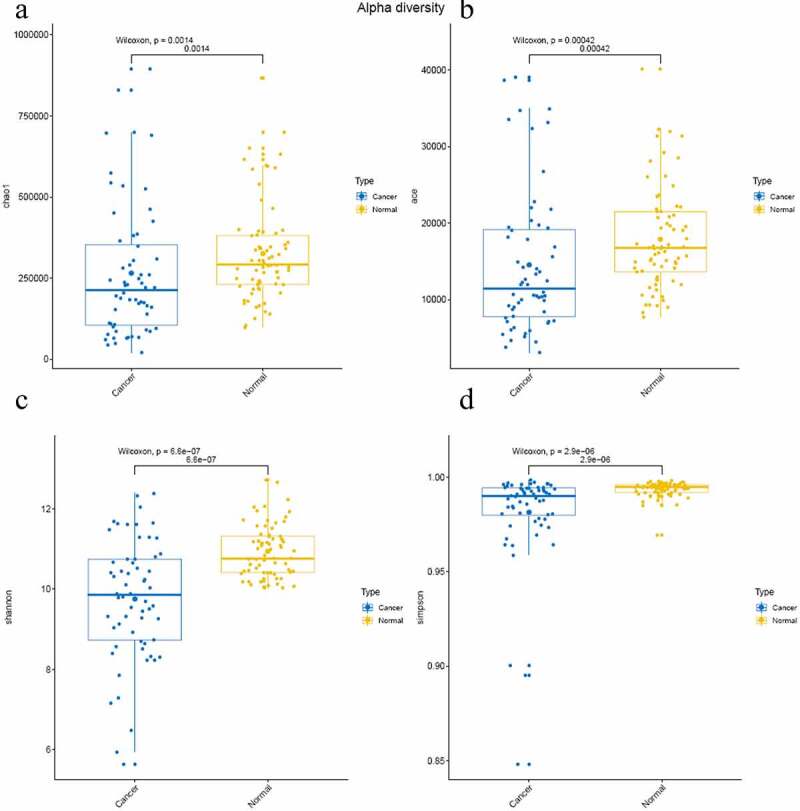
Microbial diverisity of normal group and colon cancer group

In this study, the indexes of Chao1, ACE value, Shannon index, and Good’s coverage to reflect the relative abundance and microbial diversity of different groups, which is positivly related to the abundance of species. The sequencing depth can be reflected by Good’s coverage. The data analysis results are listed in Table 1. The results show that the Chao1 index, ACE value, and Shannon index are significantly lower in cancer group (PRS011180031-PRS011190156) than that in normal group (*P*< 0.05) (Figure 2(a-c)), suggesting that the microbial diversity and abundance decreased in colon cancer group than that in normal group. Meanwhile, the simpson index was nearly 1.0, indicating the credibility of the sequencing in this study (Figure 2(d)).

**Table 1.** The difference in the microbial diversity for various samples

Sample	chao1	ace	shannon	simpson	Goods coverage
PRS003180203	465,532.482	31,924	12.6546204	0.998110428	0.517772971
PRS003180213	233,719.2382	12,673	10.73463684	0.994446881	0.600833557
PRS003180286	218,107.1037	11,307	10.19166246	0.991549818	0.662018677
PRS003180321	225,192.6257	13,631	10.44462215	0.991047173	0.629987486
PRS003180355	304,597.2356	11,533	10.74163224	0.990013975	0.521784322
PRS003180370	614,854.7737	40,135	10.74663671	0.994512319	0.730552037
PRS003180537	139,287.3146	9049	10.64491914	0.995292988	0.60261114
PRS003180630	159,341.0714	16,733	11.14302284	0.994726355	0.635094933
PRS003180719	145,550.6242	9871	10.39812098	0.995182456	0.674261084
PRS003180889	98,677.12625	8315	10.604919	0.994978796	0.65087329
PRS003181060	335,689.0135	16,263	11.50960825	0.996744076	0.545118343
PRS003181177	352,348.3471	19,105	10.4228873	0.993901395	0.688733688
PRS00318,1447	279,027.5412	14,945	10.17357908	0.992681165	0.722352376
PRS003181961	310,907.2935	19,940	10.36600926	0.990868153	0.680553187
PRS003181975	394,331.7158	21,815	10.04326352	0.969377997	0.633374844
PRS003181980	275,424.7667	14,611	11.35735838	0.995204688	0.546165055
PRS003182008	305,447.8385	12,966	10.84527414	0.989822548	0.534761779
PRS003182084	237,554.356	21,498	10.05593133	0.990499529	0.722587673
PRS003182106	215,452.8772	12,537	10.73975758	0.994750426	0.619111489
PRS003182148	392,212.1368	19,335	10.40875339	0.989710112	0.662071521
PRS003182152	215,140.7652	15,298	11.18852816	0.996322022	0.618722019
PRS003182157	539,259.7014	31,387	10.40940252	0.994409167	0.773546381
PRS003182221	269,572.96	14,898	10.03266343	0.985555227	0.707832153
PRS003182255	399,385.0083	17,332	11.73012829	0.995691872	0.466103057
PRS003182303	268,500.4111	14,102	11.22673428	0.995568743	0.563084962
PRS003182324	596,943.9703	29,174	10.98088177	0.993106574	0.641048613
PRS003182327	229,210.8485	12,013	11.14013822	0.993824702	0.50175454
PRS003182334	632,743.438	28,500	11.78733968	0.996660699	0.586240666
PRS003182406	360,660.2927	19,562	10.43784447	0.994164176	0.701685256
PRS003182420	342,385.1175	20,764	12.06769246	0.997871707	0.529787543
PRS003182434	289,743.1182	20,836	10.26613265	0.99019213	0.696341003
PRS003182435	233,227.0714	12,501	11.07709265	0.995129411	0.567871039
PRS003182436	590,320.625	24,889	11.30458562	0.9962204	0.618971009
PRS003182477	171,799.4333	10,571	10.18376273	0.985136059	0.607360984
PRS003182631	291,347.7543	21,016	11.04816524	0.995576651	0.681356767
PRS003182644	346,214.7023	23,543	10.73157013	0.994009545	0.683048135
PRS003182683	178,464.1861	9337	11.1845093	0.994690968	0.4497697
PRS003182702	242,566.7808	23,532	10.25956035	0.98795139	0.676271997
PRS003182738	171,159.4007	10,395	11.31314137	0.996557309	0.507872016
PRS003182791	699,346.3675	31,337	11.92213393	0.997340793	0.587047846
PRS003182815	397,242.3276	22,104	12.23628509	0.997470616	0.483983476
PRS003182826	298,028.243	14,381	11.34323623	0.996577767	0.563070647
PRS003182836	104,546.1355	7702	10.09838439	0.991563543	0.646650451
PRS003182872	383,627.7097	15,894	11.07484546	0.996000571	0.595564603
PRS003182918	273,324.6111	15,691	10.06970629	0.989080839	0.693608273
PRS003182944	336,169.525	16,989	10.17608775	0.991950121	0.699109379
PRS003182985	303,712.041	21,491	11.70809006	0.996623794	0.571796026
PRS003183005	584,834.4059	28,139	11.00547552	0.994660297	0.651905239
PRS003183009	164,548.1452	9225	10.04100103	0.990024562	0.60791155
PRS003183101	282,717.3834	16,653	10.09243671	0.992512437	0.73559194
PRS003183107	341,602.9459	16,861	10.76306959	0.995660996	0.651749479
PRS003183130	326,478.175	16,774	11.11482372	0.99570391	0.607054594
PRS003183140	381,536.1734	16,778	10.52628734	0.989598491	0.639678868
PRS003183141	315,178.1214	19,691	10.13093233	0.991834243	0.763657538
PRS003190020	125,035.6	10,441	10.59998128	0.995905504	0.679319997
PRS003190045	388,239.5865	19,182	10.26353288	0.992794124	0.700899414
PRS005180319	230,334.0034	11,987	10.48081691	0.994396578	0.633515696
PRS005180395	268,002.6098	16,882	11.33923989	0.996956819	0.616070338
PRS005190005	491,309.4262	23,687	11.16465673	0.994214455	0.594053693
PRS005190024	341,146.0084	16,090	11.56998129	0.99617468	0.512963141
PRS005190041	594,525.2243	26,104	11.24869466	0.994902489	0.615001161
PRS005190085	287,135.8525	15,106	10.75508195	0.993882991	0.605056694
PRS005190205	649,791.6883	26,051	12.03317707	0.996942508	0.533566315
PRS005190232	248,400.878	14,142	10.39283197	0.994458551	0.691033413
PRS005190258	346,621.5288	17,201	11.64684099	0.996632016	0.533423499
PRS011180031	64,262.16393	5197	9.887579266	0.992119657	0.64028777
PRS011180032	85,943.77103	6326	9.136546124	0.983765772	0.682406702
PRS011180035	236,804.4832	12,296	11.6171148	0.996065842	0.475207549
PRS011180036	75,722.20109	7683	7.168333758	0.964117302	0.838910134
PRS011180037	220,865.5287	15,583	9.265160222	0.980519408	0.707182431
PRS011180038	160,049.7338	7192	8.237580547	0.973594611	0.726961643
PRS011180043	425,211.5528	21,854	11.27377169	0.99567217	0.604853812
PRS011180044	94,704.85714	7269	8.304615581	0.964023195	0.786747459
PRS011180046	90,466.38746	8436	9.511839248	0.987638066	0.707146716
PRS011180047	87,005.16183	6958	8.729004523	0.980344757	0.783138419
PRS011180051	67,148.85401	4520	9.285895046	0.989475171	0.664941367
PRS011180052	20,422.1828	3086	8.634190534	0.978167601	0.754352031
PRS011180054	574,925.887	33,586	11.691358	0.996784344	0.625560803
PRS011180055	461,765.6047	19,367	11.63715721	0.996590466	0.526702133
PRS011180057	305,024.7576	20,323	12.32671211	0.998393336	0.5198093
PRS011180058	110,921.3333	7118	8.39798555	0.967591575	0.761348331
PRS011180059	66,538.80488	5551	6.48635028	0.895188023	0.831565121
PRS011180060	386,193.7268	22,783	12.04837569	0.997385202	0.550595175
PRS011180066	195,048.1481	8981	7.84724589	0.958793519	0.810856524
PRS011180067	380,468.2678	21,975	11.60208438	0.996638515	0.594575416
PRS011180068	542,793.6898	19,442	10.30752463	0.984075112	0.620418635
PRS011180069	261,120.3357	12,440	9.590069864	0.991593232	0.728287037
PRS011180070	689,919.5835	34,945	10.80145799	0.990836322	0.642764616
PRS011180072	524,984.0015	26,748	11.29199535	0.99471497	0.596826101
PRS011180078	69,914.2125	6008	10.7441622	0.993975275	0.476465028
PRS011180079	230,548	12,703	9.541590648	0.991286583	0.747672709
PRS011180102	182,731.662	10,548	11.28932894	0.990956285	0.442150151
PRS011180107	106,210.454	8734	11.62398323	0.995762233	0.398128898
PRS011190033	188,907.5636	9644	5.648068475	0.848307869	0.817487401
PRS011190034	242,943.8536	18144	10.66967616	0.995304215	0.718766478
PRS011190036	220,054.7383	11,013	10.44023202	0.993466305	0.613868777
PRS011190038	894,834.6655	33,114	12.3842141	0.996700044	0.506889275
PRS011190042	43,463.4	3777	10.41313542	0.994667764	0.503802281
PRS011190044	363,918.2683	14,926	9.810560659	0.990894205	0.71769915
PRS011190055	261,325.8482	19,705	10.2309785	0.993050148	0.749631832
PRS011190057	828,643.5782	39,055	10.44550063	0.989724884	0.666408476
PRS011190087	172,577.6552	10,504	9.445859714	0.98877041	0.747144422
PRS011190088	534,431.2091	34,741	10.1048923	0.986772659	0.754376529
PRS011190090	153,774.5263	10,731	7.296319463	0.963680301	0.875148302
PRS011190092	233,955.0833	13,654	9.691413105	0.987397556	0.693517499
PRS011190093	139,194.6857	9865	10.02947153	0.992555857	0.698778697
PRS011190094	64,136.88125	4691	5.943457229	0.900246435	0.852789308
PRS011190095	290,903.3881	17,858	8.927755101	0.985846057	0.810062447
PRS011190096	177,124.537	10,329	10.20108653	0.994370239	0.692340108
PRS011190097	108,865.7355	9253	9.034766948	0.988546944	0.807585934
PRS011190098	174,626.4929	10,353	8.239010603	0.97473656	0.801795495
PRS011190100	101,084.0058	6034	8.572465559	0.974370161	0.661542114
PRS011190106	698,727.9804	32,328	10.52078432	0.992513509	0.683749309
PRS011190121	205,549.1195	10,000	10.74579764	0.994243261	0.561882572
PRS011190123	349,343.9669	13,392	8.703346137	0.976401474	0.75809083
PRS011190124	280,153.563	11,921	9.310458486	0.98105931	0.697755904
PRS011190131	49,762.4931	7794	9.788545863	0.984604568	0.721850352
PRS011190137	249,747.2893	14,001	8.508122284	0.96958457	0.776507969
PRS011190138	228,450.4482	14,255	10.38632021	0.99347063	0.655905654
PRS011190139	309,862.6235	16,893	10.88363999	0.993927705	0.633315519
PRS011190142	184,181.2813	11,978	10.64687095	0.994255115	0.643379971
PRS011190145	450,580.0627	19,078	9.889177666	0.990139036	0.711366884
PRS011190153	697,677.665	38,633	11.47743836	0.996319024	0.677486409
PRS011190156	60,483.30556	5459	9.322985272	0.980718268	0.683848797
PRS011190159	165,174.5691	10,533	8.324783521	0.977780258	0.783931443
PRS016180405	866,770.4219	32,273	12.70927509	0.997247377	0.446479577
PRS016180416	284,157.4159	18,017	10.95751084	0.994308708	0.600912469
PRS016180421	251,442.5074	14,713	11.17862028	0.994730646	0.555822521
PRS016180432	246,327	15,085	11.5781907	0.995493445	0.510062937
PRS016180448	201,571.8341	9309	11.95532019	0.99781867	0.336496787
PRS016180483	180,284.4124	13,731	10.45884079	0.987114267	0.613306562
PRS016180493	203,891.1202	15,445	10.63845888	0.990891798	0.618039882
PRS016180503	322,787.6685	22,210	11.67960605	0.99557296	0.535865728

### 3.2. The composition analysis of gut microbes

The composition of the gut microbes in excrement of normal group and colon cancer group is analyzed based on the levels of phylum, class, genus and species, which was according to the sequencing data.

#### 3.2.1. Phylumbased microbial communities analysis

The phylum-based comparative microbial communities analysis was analyzed (Fig. S2A). The results indicated that the most dominant phylum in cancer and normal groups are Bacteroidetes, Fermicutes, respectively, and the abundances of phylums of Fermicutes, Bacteroidetes in cancer group are significantly down-regulated and up-regulated compared with normal group, respectively. And the abundances of the phylums of Proteobbacteria and Fusobacteria were also significantly up-regulated in cancer group compared with normal group (*P*< 0.05). The abundances of Classes including Clostridia, Bacteroidia, and Negativicutes are the highest in normal group, whereas classes including Clostridia, Bacteroidia, and Baccilli are the highest in colon cancer group.

#### 3.2.2. Class and order based microbial communities analysis

According to the class-based comparative microbial communities analysis (Fig. S2B), the class of Clostridia was significantly less in cancer group than that in normal group (*P*< 0.05). Meanwhile, the abundances of the classes including Negativicutes, Gammaproteobacteria, Bacilli, Actinobacteria are significantly higher in cancer group than that in normal group (*P*< 0.05). As shown in Fig. S2C, the abundances of orders including Clostridiales, Bacteroidales and Selenomonadales are the highest in normal group, whereas classes of Clostridiales, Bacteroidales and Lactobacillales are of the most abundance in cancer group. The Clostridiales class is significantly lower in colon cancer group, and the classes of Selenomonadale, Enterobacteriales, and Lactobacillales are significantly up-regulated in colon cancer group than that in normal group.

#### 3.2.3. Genus and species-based microbial communities analysis

**Figure 3.**
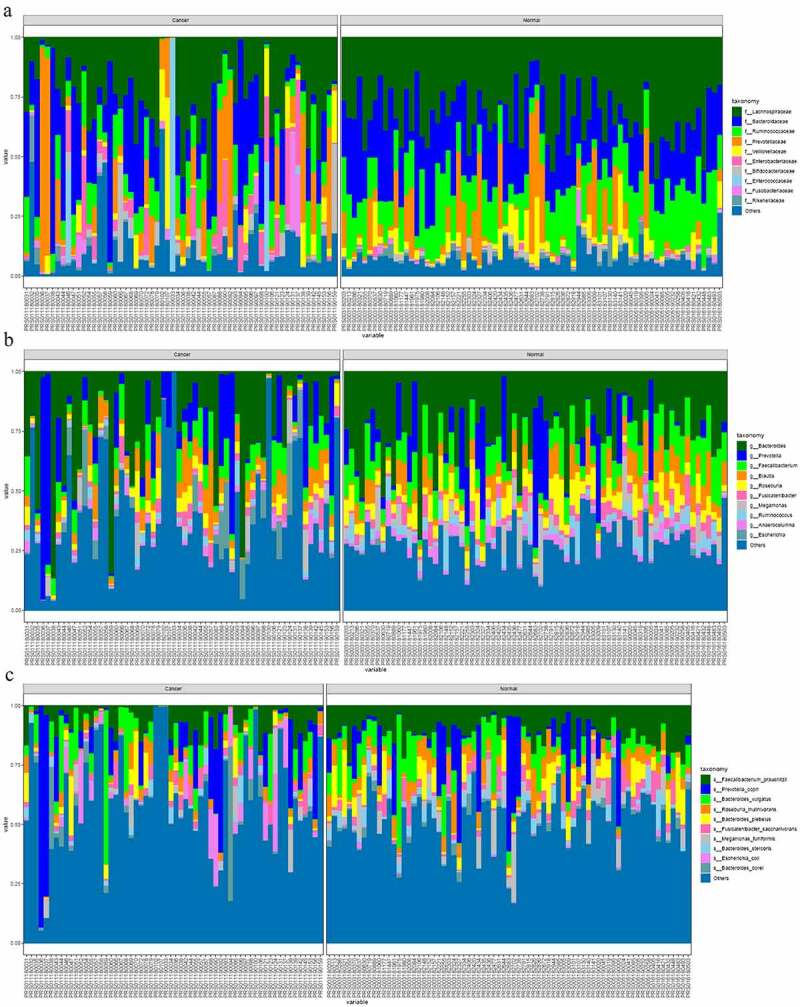
The microbial difference in normal group and colon cancer groups based on family (a); genus (b) and species (c)

The comparative map for the different microbial communities in normal and cancer groups was depicted. The family-based differential map indicated that the abundance of the families of Lachnospiraceae, Bacteroidaceae, and Ruminococcaceae are significantly down-regulated in colon cancer group. And families including Prevotellaceae, Veillonellaceae, and Enterobacteriaaceae are significantly higher in cancer group than that in normal group (Figure 3(a)). As shown in Figure 3(b), the most dominant genus in cancer group and normal group are Bacteroides, Prevotelia, Faecalibacterium, and Blautia. And the genus of Bacteroides, Faecalibacterium, and Roseburia in colon cancer group are significantly higher in normal group than that in normal group. And genus of Prevotella and Blautia in colon cancer group are significantly higher than that in normal group. The comparative species map of the two groups were depicted. The dominant species in the two groups are *Faecalibacterium prausnitzii, Prevotella copri*, and *Bateroides vulgatus*. The species of *Faecalibacterium prausnitzii, Bateroides vulgatus,* and *Fusicatenibacter saccharivorans* are significantly lower in cancer group than that in normal group (*P*< 0.05). The species of *Prevetella copri* and *Escherichia coli* are significantly higher in cancer group than that in normal group.

Beneficial bacteria including *Bifidobacterium adolescent, Bifidobacterium Longum, Faecalibacterium prausnitzii, Roseburia faeci,* and *Fusicatenibacter Scharivorans* were involved in the synthesis and consumption of neurotransmitters, and the contents of some microbial neuroactive metabolites also increased significantly. The intake of these beneficial bacteria can relieve the stress of the subjects. The contents of these beneficial species were significantly decreased in the colon cancer group compared with the normal group.

### 3.3. The heatmap analysis

The heatmap based on different levels between cancer group and normal group is depicted. The heatmap based on phylum showed that the phylum of Firmicutes, Bacteroidetes, Proteobacteria, and Actinobacteria showed significant difference (*P*< 0.05). And partly samples of the two groups also showed significant difference (Fig. S3A). As shown in Fig. S3B, classes including Negativicutes, Clostridia, Bacteroidia, Gammaproteobacteria, Bacilli, Actinobacteria, Betaproteobacteria, and Erysipelotrichia showed significant difference between the cancer group and normal group. And the order of Selenomonadales, Clostridales, and Bacteroidales showed the most significant difference between the two groups (Fig. S3C). And partly samples from the two groups also showed significant difference in the order of Enterobacteriales, Bifidobacteriales, Lactobacillales, Coriobacteriales, B urkholderiales, and Erysipelotrichales.

**Figure 4.**
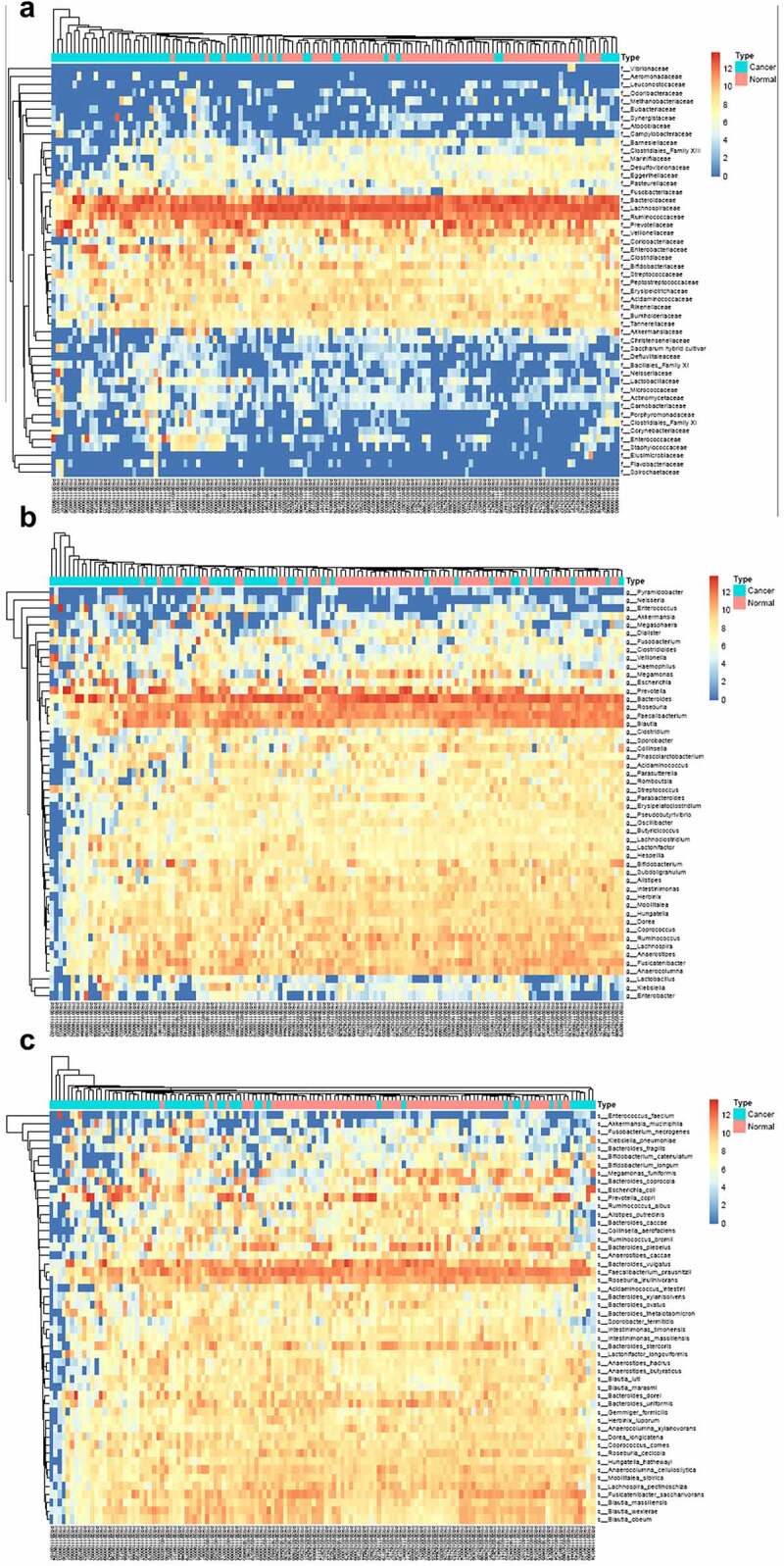
The heatmap between the normal and colon cancer group based on different levels

The abundance of the family of Bacteroidaceae, Lachnospiraceae, and Ruminococcaceae in the normal group and cancer group showed very significant difference (*P*< 0.01), and the abundances of Prevotellaceae, Veillonellaceae, Coriobacteriaceae, Enterobacteriaceae, Clostridiaceae, Bifdobacteriaceae, Streptococcaceae, Peptostreptococcaceae, Eryipelotrichaceae, Acidaminococcaceae, Rikenellaceae, Burkholderiaceae, Tannerellaceae are relatively high (Figure 4(a)). The genus differential map indicated that the genus of *Prevotella, Bacteroides, Roseburia, Faecalibacerium, Blautia* showed very significant difference in normal group and cancer group (*P*< 0.01), meanwhile, the abundance of genus of *Clostridium, Sporobacter, Colinsella, Phasolarctobacterium, Acidaminococcus, Parasutterella, Romboutsia, Streptococcus, Parabacteroides, Erysipelatoclostridium, Pseudobutyrivibrio, Oscillibacter, Butricicoccus, Lachnoclostridium, Lactonifactor, Hespellia, Bifidobacterium, Subdoligranulum, Alistipes, Intestinimonas, Herbinix, Mobilitalea, Hungatella, Dorea, Coprococcus, Ruminococcus, Lachnospira, Anaeostipes, Fusicatenibacter*, and *Anaerocolumna* showed a significant difference (*P*< 0.05) (Figure 4(b)). And the abundance of the species of *Megamonas funiformis, Bateroides coprocola, Escherichia coli, Prevotella copri, Ruminococcus albus, Alistipes putredinis, Bacteroides caccae, Collinsela aerofaciens, Ruminococcus bromii, Bacteroides plebeius, Anaerostipes caccae, Bacteroides vulgatus, Faecalibaterium prausnitzii, Roseburia inulinivorans, Bacteroides stercoris, Bacteroies dorei, Bacteroides uniformis, Gemmiger formicilis, Herbinix luporum, Anaerocolumna xylanovorans, Dorea longicatena, Coprococcus comes, Roseburia cecicola, Anaerocolumna cellulosiltica, Lachnospira pectinoschiza, Fusicatenibacter saccharivorans, Blautia massiliensis, Blautia wexlerae*, and *Blautia obeum* showed very significant difference between the two groups (*P*< 0.01), the abundances of the species of *Enterococcus faecium, Akkermansia muciniphila, Fusobacterim necrogenes, Klebsiella pneumoniae, Bacteroides fragilis, Bifidobacterium catenulatum*, and *Bifidobacterium longum* did not show significant difference in the groups (Figure 4(c)) .

*Prevotella copri* is strictly an anaerobic, which is extremely sensitive to oxygen and can only grow well completely in an anaerobic environment. It can metabolize polysaccharide such as Xylan, also can metabolize small molecular sugar such as hemicellulose, xylose. The development of cancer in colon results in the condition of low-oxygen and high-concentration of sugar, which facilitate the growth of *P. copri*. Higher levels of *P. copri* were also detected in patients with rheumatoid arthritis and psoriatic arthritis [28–30].

*M. uniformis* has the potential to prevent and/or treat inflammation-related diseases such as digestive tract inflammation-related diseases such as ulcerative colitis, gastritis and gastroenteritis, as well as cardiovascular diseases such as inflammatory bowel disease rheumatoid arthritis. Thus, the colon cancer in the patients leads to the significant decrease of in the intestinal of *M. uniformis* patients suffered from colon cancer. It is true that there are significant differences in the gut microflora between gouty patients and healthy people. The gut bacteria of gouty patients are rich in bacteria such as *Bacteroides caccae* and *Bacteroides xylanisolvens*, while the other two species (*Faecalibacterium prausnitzii* and *Bi dobacterium pseudocatenulatum*) are absent in patients suffered from gouty [31–34]. The results indicated that the genus of Bacteroides are beneficial bacterial for patients, and genus of Faecalibacterium and Bidobacterim are harmful for colon cancer patients.

### 3.4. Intergroup similarity analysis

PCOA (PCOA) is a kind of visualization method to study the similarity or difference of multi-dimensional data, which was used to investigate the similarity of microbial communities between normal group and colon cancer group.

**Figure 5.**
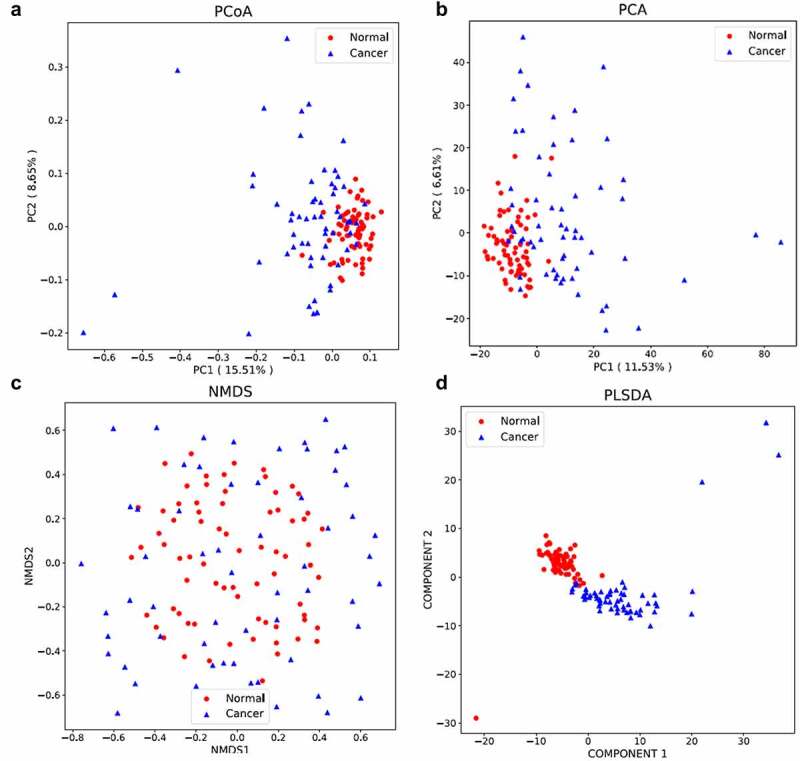
The significance differential analysis of the normal and colon cancer groups

PC1 and PC2 represent the first principal component and the second principal component, respectively, and the percentage after the principal component represents the contribution rate of this component to the sample difference. The distance of the sample points represents the similarity of the functional classification distribution in the samples. The results suggested that high similarity of the samples in the normal group, whereas great difference was observed in the samples from colon cancer group and the samples from the different groups. PC1 and PC2 contributed 15.51% and 8.65% to the difference between the two groups (Figure 5(a)).

Principal component analysis is a technique to simplify the analysis of data, which can effectively identify the dominant elements and structures in the data. The similarity and difference among samples can be reflected by analyzing the distribution of bacterial community in different samples (Figure 5(b)). PC1 and PC2 contributed 11.53% and 6.61% to the difference between the two groups.

NMDS (non-metric multidimensional scaling) reflected in the multi-dimensional space in the form of points, and the degree of difference between different samples according to the species information contained in the sample. The NMDS analysis is shown in Figure 5(c). And the distribution of colon cancer group is more disperse than that in normal group.

Partial least squares discrimination analysis (PLS-DA) is a multivariate statistical analysis method for discriminant analysis. Discriminant analysis (DA) is a common statistical analysis method to determine the classification of research objects according to the observed or measured values of several variables. The principle of this method is to train the characteristics of different treatment samples (such as observation samples and control samples), to generate training sets, and to test the credibility of training sets. The PLS-DA is analyzed in Figure 5(d), and the distribution of the samples was not so disperse, indicating the reliability of the sequencing results.

### 3.5. The biological correlation analysis

**Figure 6.**
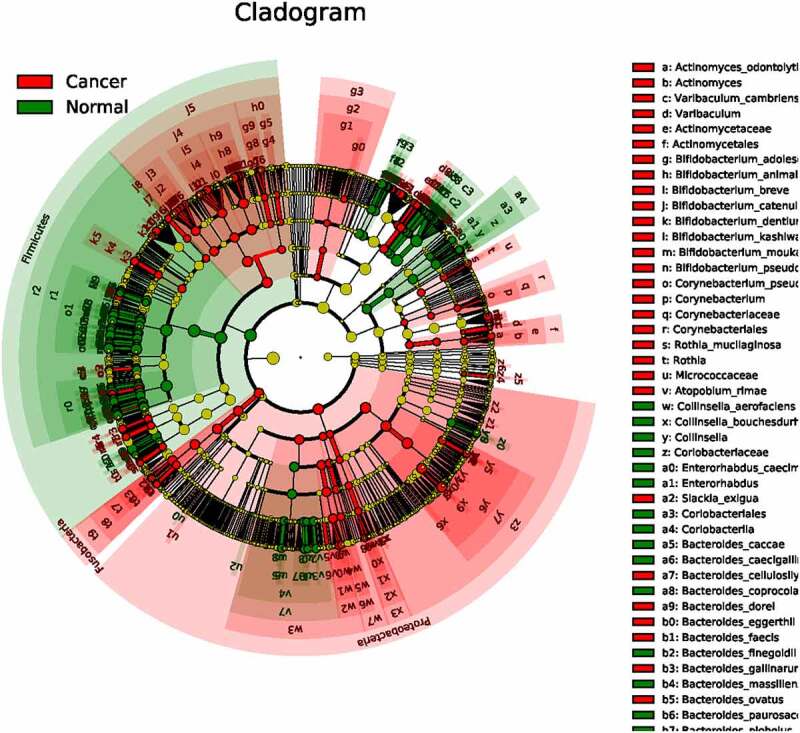
The cladogram analysis of the normal and colon cancer group

The UPGMA analysis of the normal and cancer group indicated the significant difference in the microbial communities (Fig. S4). LDA effect size analysis is an analysis tool for discovering and interpreting biomarkers of high latitude data. This method emphasizes statistical significance and biological correlation, and can discover biomarkers with statistical differences between groups. As shown in Figure 6(a), the most dominant bacterial communities include Clostridales, Clostridia, Firmicutes, Lachnospiraceae, Ruminococcaceae, Facalibacterium, and the most dominant species is *Facalibacerium prausnitzii* in normal group, species including *Roseburia inulinivorans, Bacteroides plebeius,* and *Megamona funiformis* took the second to the fourth places in the normal group. The most dominant bacteria communities in cancer group include Proteobacteria, Bacilli, Lactobacillales, Gammaproteobacteria, Enterobacteriales, Enterobacteriaceae, and Enterococcaceae. And the most dominant species in colon cancer group is *Escherichia coli*, followed by *Bacteroides dorei, Enterococcus faecium, Neisseria mucosa, Bacteroides ovatus,* and *Bifidobacterium catenulatum*.

Proteobacteria are the largest group of bacteria, including many known pathogens such as *E. coli, Salmonella, Vibrio cholerae*, and *Helicobacter pylori*. There are also free-living species, including many nitrogen-fixing species. Bacteroides are Gram staining negative bacteria with the features of non-spore-forming, obligate anaerobic bacillus. Bacteroides normally inhabiting in the intestine, oral cavity, upper respiratory tract, and reproductive tract of humans and animals. Due to the long-term use of broad-spectrum antibiotics, hormones, immunosuppressants, bacteroides can cause the body immune function disorders or dysbacteriosis, leading to endogenous infection. Bacteroides can decompose peptone or glucose to produce succinic acid, acetic acid, formic acid, lactic acid, and propionic acid, thus facilitating the growth and transfer of colon cancer cells [33,34].

The cladogram between the normal group and colon cancer group was also depicted. As shown in Figure 6(b), the radiations from inner to outer of different circles represented seven taxonomic levels of Phylum, family, genus and species, and each node represented a species classification at that level. The yellow node color indicates that the species has no significant difference in the comparison group, if the node color is red, the species has significant difference in the comparison group (*p < 0.05*). The results showed that most significant different species between the two groups belong to proteobacteria phylum, and the least most significant different species between the two groups belong to firmicutes phylum.

## 4. Conclusions

In this study, excrement from the healthy crowd and patients suffered from the colon cancer were sequenced. The significant microbial communities based on levels of phylum, class, order, family, genus, and species were analyzed using comparative composition analysis and heatmap. The phylum of Fermicutes, Bacteroidetes in cancer group are significantly down-regulated and up-regulated compared with normal group. The species including *Faecalibacterium prausnitzii, Bateroides vulgatus,* and *Fusicatenibacter saccharivorans* are significantly lower in cancer group than that in normal group (*P*< 0.05), suggesting that the complement of these species would be beneficial for colon cancer patients. The species of *Prevetella copri, M. uniformis,* and *Escherichia coli* are significantly higher in cancer group than that in normal group. The comparative results indicated that some beneficial bacterium significantly decreased in cancer group, and some harmful bacterium significantly increased in colon cancer group, which maybe due to the increased acidity, sugar and decreased oxygen content in colon cancer cells. The results would provide mirobial approaches for the treatment of colon cancer by the intake of beneficial microbial communities.

## Supplementary Material

Supplemental Material

## Highlights

1. The microbial diversity of the faecal from normal crowds and colorectal cancer patients were analyzed;
2. Species including *Faecalibacterium prausnitzii, Bateroides vulgatus* are significantly lower in CRC group;
3. The results indicated the role of some gut microbial for the development of CRC;
4. This study would offer microbial clues for the prevention and treatment of CRC.
