# Supplementary figures and images for: The gut microbial diversity of colon cancer patients and the clinical significance
Source: Bioengineered. 2021 Sep 23;12(1):7046–60. doi: 10.1080/21655979.2021.1972077 (PMC8806656; doi:10.1080/21655979.2021.1972077)

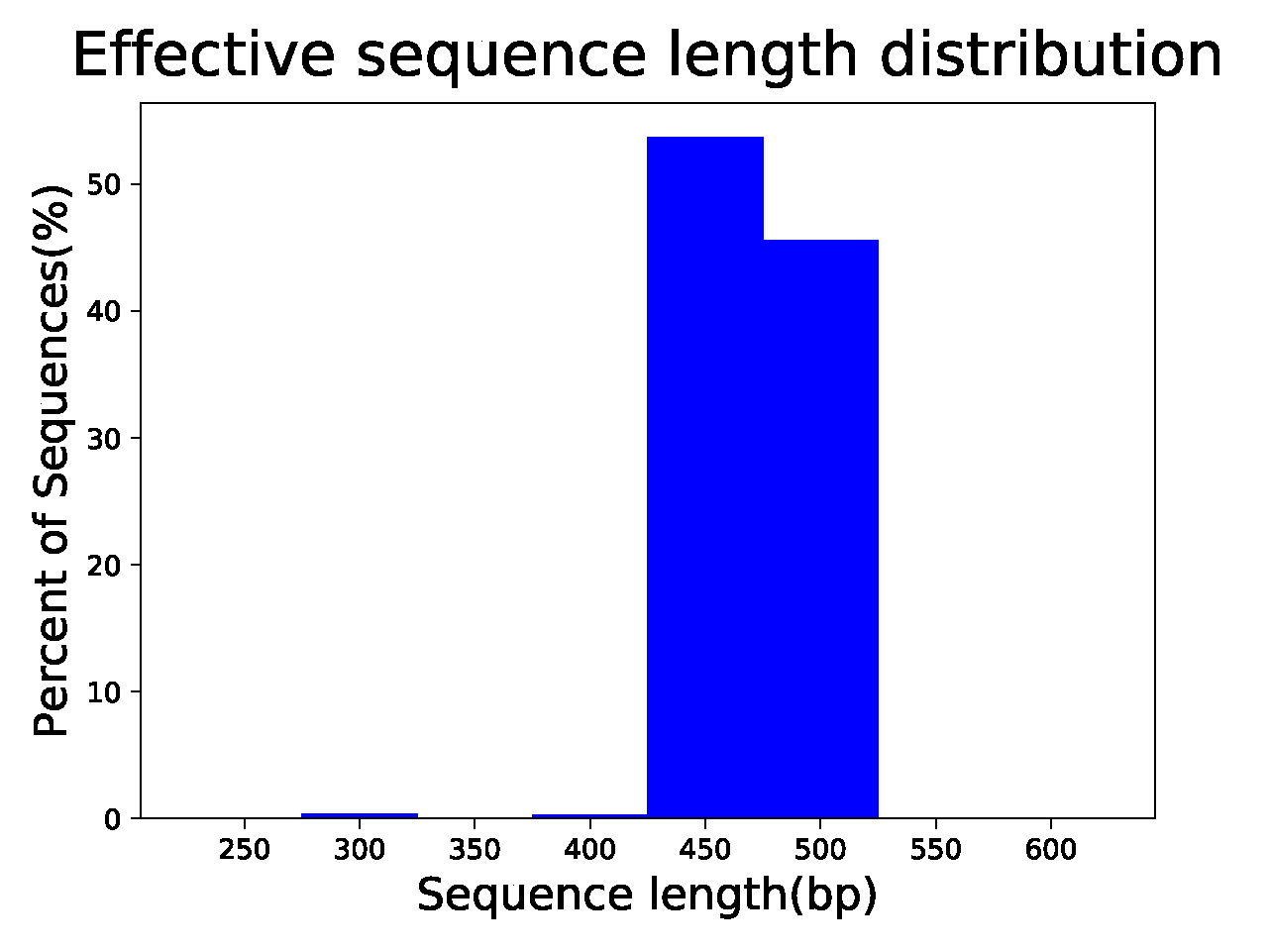

Supplement: Supplemental Material [file KBIE_A_1972077_SM2795.zip › supplementary/figure S1 (1).tif]

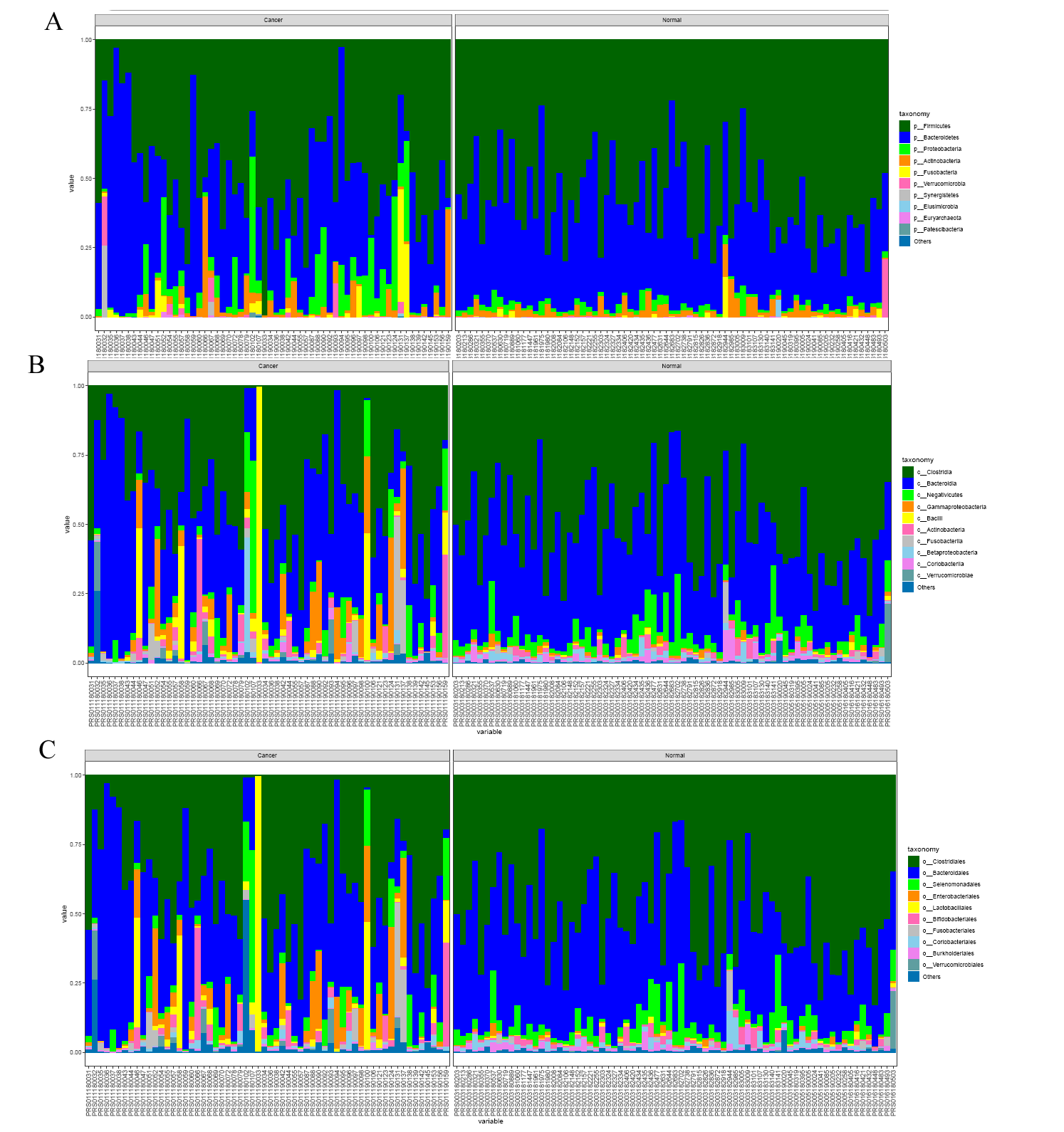

Supplement: Supplemental Material [file KBIE_A_1972077_SM2795.zip › supplementary/figure S2.tif]

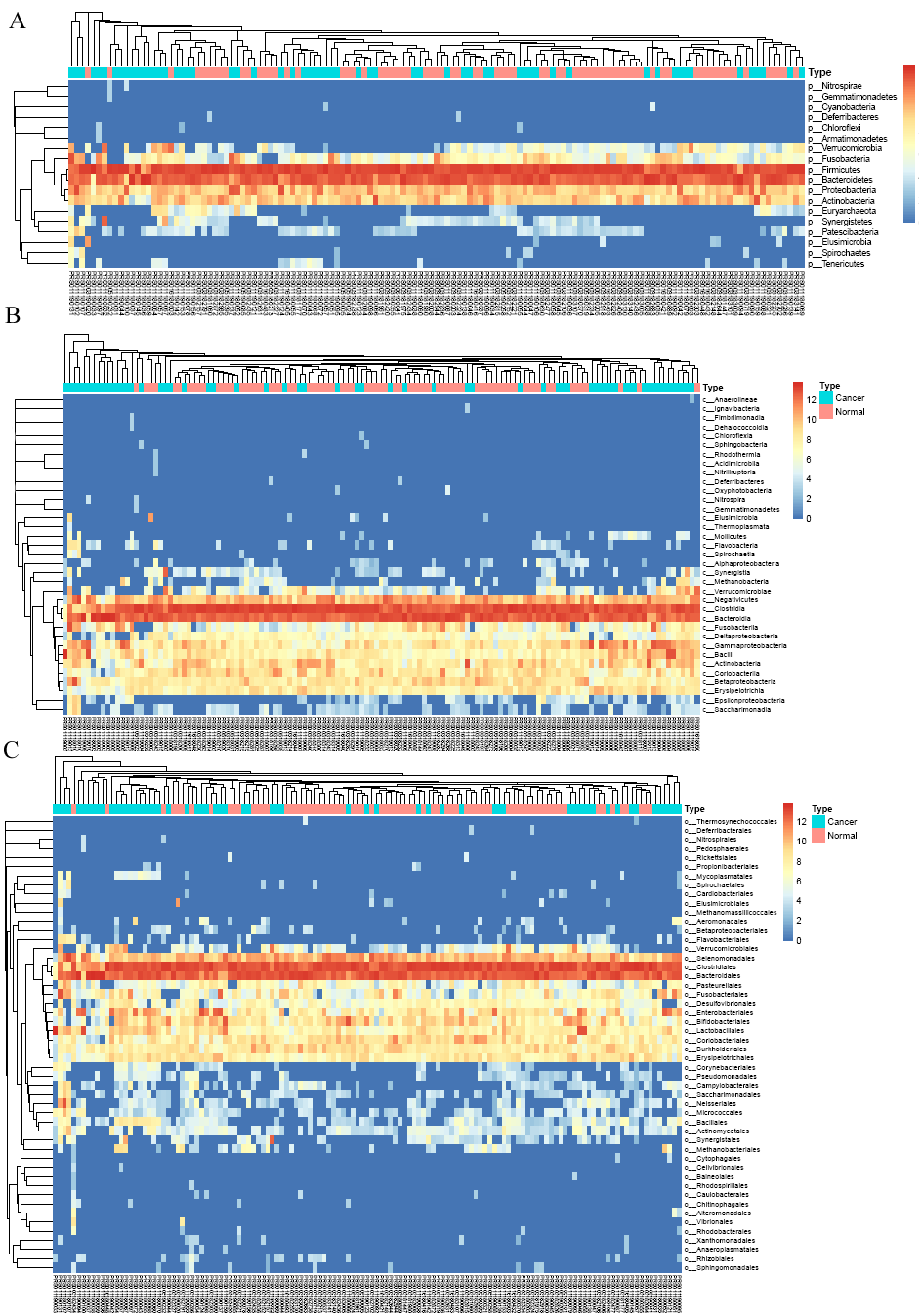

Supplement: Supplemental Material [file KBIE_A_1972077_SM2795.zip › supplementary/figure S3.tif]

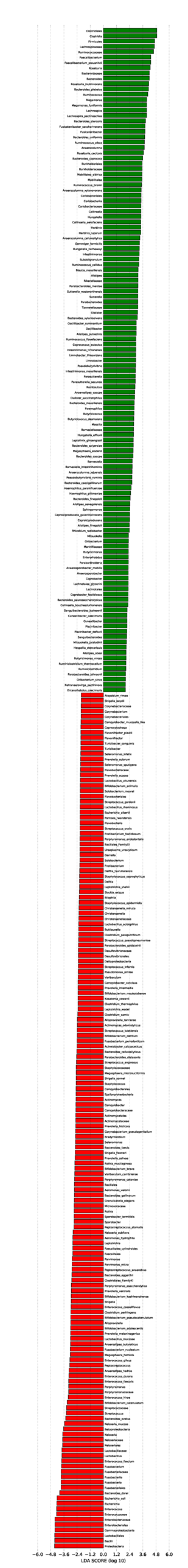

Supplement: Supplemental Material [file KBIE_A_1972077_SM2795.zip › supplementary/figure S4.tif]
